# Editorial: Search of Individually Optimal Movement Solutions in Sport: Learning Between Stability and Flexibility

**DOI:** 10.3389/fpsyg.2021.728375

**Published:** 2021-08-09

**Authors:** Ana F. Silva, John Komar, Ludovic Seifert

**Affiliations:** ^1^N2i, Polytechnic Institute of Maia, Maia, Portugal; ^2^The Research Centre in Sports Sciences, Health Sciences and Human Development (CIDESD), Vila Real, Portugal; ^3^National Institute of Education, Nanyang Technological University, Singapore; ^4^Cetaps EA3832, Faculty of Sport Sciences, University of Rouen, Normandie, France

**Keywords:** flexibility, adaptability, sport, skill learning, ecological approach, constraints

## Introduction

We have always been fascinated by how complex skills are learned and stabilized by experts. Although motor learning has been seen for long merely as a process of stabilization of an optimal solution, it has been recently described that many pathways could be outlined to attain expertise in sports. Recent studies suggested that early specialization could lead to a lack of perceptual-motor adaptability, i.e., difficulties in how performers become attuned to affordances (opportunities for action). Thus, it has been argued that expert performance requires a subtle balance between movement stability and flexibility (Seifert et al., 2013, 2016). The ecological dynamics framework offers a rich, unifying perspective to understand and explain sports performance, providing an innovative perspective on talent development and motor learning, highlighting a nuanced transitioning between specificity and generality of practice and transfer, as needed by each individual (Button et al., 2020). This Research Topic included studies on talent development to achieve sport expertise, motor learning and interventions. It particularly explores the functional role of variability in searching for an individually optimal movement solution. Contributions were classified as: (i) variability as skill adaptation, flexibility, and discuss about adaptability, (ii) variability as individual movement solution, and (iii) variability in interventions, practice, and pedagogy.

## Variability As Skill Adaptation/Adaptability/Flexibility

The challenge in sports performance is to sort what is a “good” (functional) from “bad” (dysfunctional) variability (Latash et al., 2010). To achieve that, not only an expert movement but sports intelligence has been a central concern. Hristovski and Balagué proposed a theory of cooperative-competitive intelligence (CCI) based on: (i) relativity of functional entropy/information in agent (team) environment; (ii) tendency toward the satisficing level of diversity/uncertainty potential; and (iii) tendency toward the non-decreasing potential.

When comparing experts to non-experts, it was showed that all swimming levels were able to change the movement pattern (swim pace), but high-level swimmers exhibited a broader functional adaptation in force parameters (Schnitzler et al.). Also, in karate, it was observed that experts were unable to repeat the kinematics of a front kick movement (Burdack et al.). However, with fatigue, the short-term movement-patterns does not change, only the overall kinematic movement pattern. Indeed, Woods et al., in Australian football, highlighted the relevance to understand affordances to regulate performance behaviors, as they occur according to an ecological approach, being the skilled behavior a functionally adaptable performance solution that arise from the continuous interactions with the environment (Araújo and Davids, 2011). Those sport specific analysis allow coaches to guide learning, understanding the important parameters affecting higher performance levels. Indeed, in baseball it was found that the elevation pitching angle and speed significantly influenced the vertical pitch location, and the azimuth pitching angle significantly influenced the horizontal pitch location (Kusafuka et al.).

## Variability As Individual Movement Solution

The existence of individual movement responses has been strongly identified as a hallmark of skilled performance and learning with the growing emphasis of the constraints model from Newell (1986). This aspect has been recently emphasized due to advancements in data analytics that can handle large and multivariate data set and can account for both inter- and intra-individual differences in movement behavior. Using a support vector machine technique, Horst et al. effectively identified a strong individual component in throwing patterns. This is highlighted in various throwing disciplines, although at different degrees depending on the discipline (e.g., stronger individuality in shot put and discus than in javelin). This observation is discussed also by Ranganathan et al. highlighting that different sport skills have dissimilar demands for behavioral flexibility. Athletes with greater flexibility are capable of showing more diverse movement solutions, therefore would be more likely to find his/her own optimal individual solution. However, too much flexibility may impair performance if the task or environmental constraints are less dynamic.

Ranganathan et al. propose to revisit the famous quote from Bernstein (1967) “repetition without repetition” to highlight the key role of movement flexibility in behavioral adaptability but also for learning. In that view, an optimal movement solution can actually refer to an optimal level of movement flexibility (i.e., in addition to the more common consistency and efficiency criteria). This is highlighted by Fernández-Valdés et al. who identified that the increase in performance during a 6 weeks practice appeared during a plateau of variability, somehow during an optimal movement variability, before the task constraints become too predictable therefore not requiring adaptive flexibility anymore. This result precisely highlights a key moment in training and learning when the task constraint may need to evolve to challenge again flexibility of individual movement solutions.

## Variability in Intervention, Practice, and Pedagogy

Three different forms of variability could be induced during pedagogical intervention: intrinsic, structured and unstructured (Ranganathan and Newell, 2013). During constant practice, variability is intrinsic to motor system, but often insufficient for learners to leave their initial stables states. Thus, structured variability could be used to guide perceptual attunement, so less useful information becomes unreliable during learning (Fajen and Devaney, 2006), resulting in better performances in transfer tasks instead of constant practices (Huet et al., 2011). Schöllhorn et al. (2009) proposed to add unstructured variability to practice at the level of multiple task parameters. To investigate how unstructured variability can enhance motor learning, Tassignon et al. performed a meta-analytic review on the empirical evidence of differential learning. However, given the large amount of heterogeneity, limited number of studies, low sample sizes, low statistical power, possible publication bias, and high risk of bias in general, the authors concluded that inferences about the effectiveness of differential learning would be premature. Even though differential learning shows potential to result in greater average improvements between pre- and post/retention test compared to non-variability-based motor learning methods, more high-quality research is needed before issuing such a statement.

As virtual reality (VR) becomes more popular in cognitive sciences, scientists could be tempted to use it to design variable practice. In the study of Drew et al., VR training showed to impair real-world task performance, suggesting that virtual environments may offer different learning constraints. These results emphasize the need to better understand how some elements of VR environments detract from transfer of an acquired sport skill to the real world.

Otte et al. developed a Periodization of Skill Training (PoST) framework, to propose a model that aims to support practitioners' understanding of the pedagogical constraints of feedback and instruction during practice. In this “hypothesis and theory” article, the PoST framework attempted to guide practitioners on how and when to apply different verbal instruction methodologies and aim to support the design of effective skill learning environments.

In conclusion, it appears from this topic that searching for optimality of movement in sport requires the consideration of the functional role of movement variability, through the lens of flexibility and adaptability. However, looking at movement variability strongly depends on what is considered as a stable movement solution, where stability should be understood at an individual level and therefore an optimal stable movement for one athlete may not be optimal for another athlete. Then, if both reaching expertise requires to develop adaptability to dynamic environments as well as an highly individual stable solution, the path to expertise should also consider this functional role of variability in order to facilitate the search for an individually optimal but adaptable motor solution. Looking at this perspective where functional variability plays a role both in the outcome of learning as well as in the process of learning opens a renewed view on key topics in movement and sport science. For instance, could adaptability of athletes better predict future performance, to inform talent identification. Another key direction relates to injury prevention; Could training methods that promote the infusion of variability better prevent injuries?

## Author Contributions

All authors contributed in the editing the papers received as well as in the Editorial manuscript, both in conceptualization and in writing.

## Conflict of Interest

The authors declare that the research was conducted in the absence of any commercial or financial relationships that could be construed as a potential conflict of interest.

## Publisher's Note

All claims expressed in this article are solely those of the authors and do not necessarily represent those of their affiliated organizations, or those of the publisher, the editors and the reviewers. Any product that may be evaluated in this article, or claim that may be made by its manufacturer, is not guaranteed or endorsed by the publisher.
